# Rise and Fall of Physical Capacity in a General Population: A 47‐Year Longitudinal Study

**DOI:** 10.1002/jcsm.70134

**Published:** 2025-11-16

**Authors:** Maria Westerståhl, Gustav Jörnåker, Eva Jansson, Ulrika Aasa, Michael Ingre, Kaveh Pourhamidi, Brun Ulfhake, Thomas Gustafsson

**Affiliations:** ^1^ Department of Laboratory Medicine, Division of Clinical Physiology Karolinska Institutet Stockholm Sweden; ^2^ Unit of Clinical Physiology Karolinska University Hospital Stockholm Sweden; ^3^ Medical Allied Health Professionals Karolinska University Hospital Stockholm Sweden; ^4^ Department of Medicine Karolinska Institutet Stockholm Sweden; ^5^ Department of Clinical Neurophysiology Karolinska University Hospital Stockholm Sweden

**Keywords:** exercise test, healthy aging, longitudinal studies, middle aged, physical fitness, sarcopenia

## Abstract

**Background:**

As we age, there is a progressive decline in skeletal muscle tissue and function that can become clinically significant in the sixth decade of life affecting independent living and health. Longitudinal observations in elite athletes show that peak physical performance is reached before the age of about 35 years despite continuous training, suggesting that the tissue processes underlying muscle dysfunction may begin decades before they become clinically relevant. To answer the question of whether the pattern of performance decline in athletes also applies to the general population, a population‐based longitudinal study is needed.

**Methods:**

In the Swedish population cohort (SPAF), 427 individuals (48% women) born in 1958 underwent repeated objective assessments of physical capacity from age 16 to 63 years. Linear mixed models were used to estimate age‐ and sex‐specific changes in the original cohort during the study period.

**Results:**

The estimated maximal aerobic capacity and muscular endurance (bench press repetitions) peaked at ages 26–36 in both sexes and declined gradually, starting at 0.3%–0.6% per year and accelerating to 2.0%–2.5% per year (main effect of age *p* < 0.001 and sex *p* < 0.01), with no sex difference in decline rates. Muscle power was measured using the Sargent jump test, with men having their peak at age 27, and women at age 19. Group variance in physical performance increased markedly with age, with relative aerobic capacity showing a 25‐fold increase, jump height a nearly 5‐fold increase, and muscular endurance a threefold increase in variance from adolescence to age 63. The rate of decline was small initially (0.2%–0.5% per year) but increased with age (2.2% per year), in both sexes (main effect of age *p* < 0.001 and sex *p* < 0.001), with no difference between the sexes. The overall decline in physical capacity from peak to age 63 ranged from 30% to 48%. Higher leisure‐time physical activity at age 16 and becoming active in adulthood were associated with better performance across all outcomes (*p* = 0.00–0.02); having a university degree was positively associated with absolute aerobic capacity (*p* = 0.04) and muscular endurance (*p* = 0.02).

**Conclusions:**

The Swedish population cohort SPAF shows the same pattern of changes in physical capacity in adulthood as previously demonstrated for elite athletes. This confirms the concept that a decline in physical capacity can be observed before the age of 40, which can later lead to clinically significant physical dysfunction, especially in individuals with a sedentary lifestyle.

**Trial Registration:** ClinicalTrials.gov identifier: NCT06496204.

## 1 Introduction

With increasing age, there is a progressive decline in tissue function and integrity known as aging [1]. When aging affects the skeletal muscle, the loss of function can result in an inability to move and balance the body during activities of daily living. This age‐related condition (ICD‐10‐CM code M62.84, sarcopenia) [2], occurs clinically in the 6th to 7th decade of life with an annual increase in prevalence of ≥ 0.5% [2]. The causes of sarcopenia and the time at which the decline in tissue function begins are still controversial [2].

To date, the only evidence‐based intervention that can at least delay the onset and progression of sarcopenia is physical activity, although skeletal muscle appears to be less adaptive in old age than in younger adulthood [3, 4]. It seems plausible that combating sarcopenia through appropriate exercise interventions could be more effective if initiated before the onset of the underlying processes. However, even elite athletes who continue to exercise throughout their life lose physical function as they age. For most athletes, peak performance is passed at ~35 years of age. This is followed by a gradual decline in performance that accelerates at older ages (annual loss of 1%–2%, ≥ 70 years) [5, 6, 7, 8, 9, 10]. In addition, some studies suggest that the slope of decline in performance may differ between men and women [11, 12]. The advantage of studying elite athletes is that longitudinal performance data with high granularity is available and that the decline is less influenced by physical inactivity. The obvious disadvantage is that elite athletes may not be representative of the population in general. To learn how physical capacity declines with age, differs between sex and is affected by physical activity/lifestyle in a general population, a longitudinal assessment of physical capacity from youth to old age is needed. If we study the individual trajectories of change in physical capacity over the course of life in a general population, physical activity recommendations can be optimized to prevent or delay sarcopenia [13].

The main aim of this study was to assess objectively measured physical capacity from adolescence to older age. We used the population‐based Swedish longitudinal cohort for Physical Activity and Fitness (SPAF), born in 1958 [14], to analyse the pattern of changes in objectively measured aerobic, muscular endurance and power ability in women and men, starting in adolescence (16 years old; 1974) to early old age (63 years old; 2021). A second aim was to assess any sex differences and, thirdly, to assess the influence of leisure‐time physical activity and educational level on physical capacity throughout life.

## 2 Methods

### 2.1 Participants

A detailed description of the selection, representativeness and drop out from the original cohort can be found in Data S1 (section Participants and drop‐out, section Representativity, Table S1 and Figure S1). In short, in 1974, 222 male and 205 female pupils aged 16 years were randomly selected to provide a representative sample of Swedish adolescents at upper secondary school [S1]. Five data collections have so far been conducted on this sample, at ages 16, 27, 34, 52 and 63 [S2‐S6, 14].

### 2.2 Measurements

#### 2.2.1 Anthropometry

**FIGURE 1:**
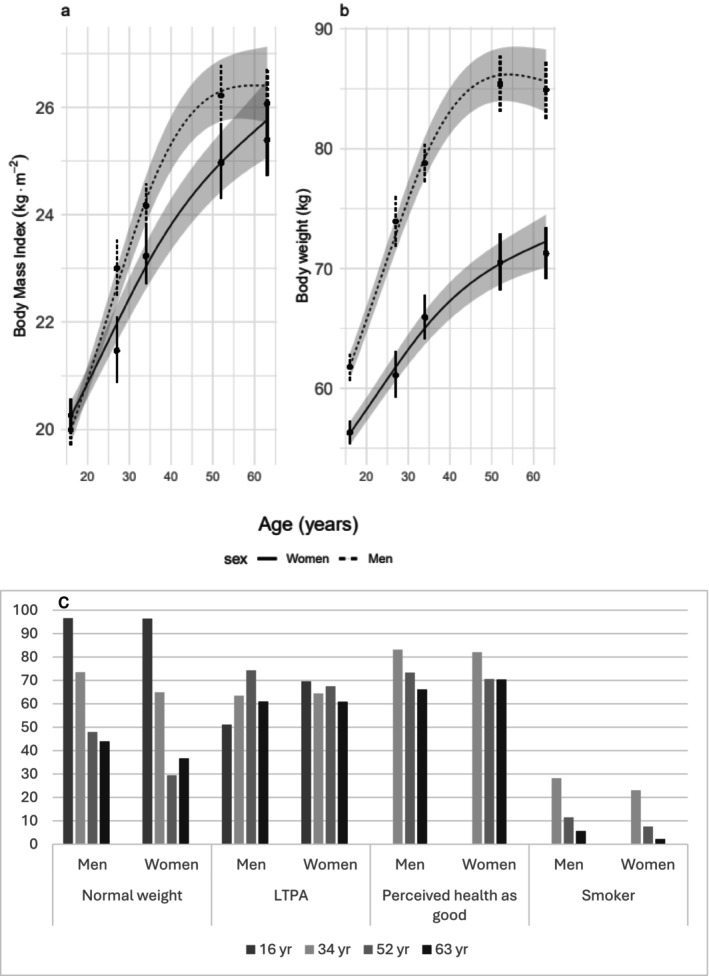
Health and Lifestyle indicators from 16 to 63 years. (a) Estimated body mass index (kg·m^−2^) from the optimised linear mixed model. (b) Estimated weight (kg) from the optimised mixed linear model. (c) The *y*‐axis represents the percentage of participants who (1) have a BMI < 24.5 form boys and < 24.7 for girls at age 16 and BMI < 25 at the older ages, (2) engage in leisure‐time physical activity (replied yes (yes/no)), (3) perceive their health as good on a 3‐grade scale (bad, either good or bad, good) and (4) are smokers (replied yes (yes/no)). LTPA = leisure‐time physical activity.

Height and weight were measured at all ages without shoes and in light sports clothing. Body mass index (BMI, kg·m^−2^) was calculated (Figure 1). Normal weight at age 16 was defined as a BMI below 24.5 for boys and 24.7 for girls [S7]. Normal weight from age 18 was defined as a BMI below 25.0 kg·m^−2^ for both sexes.

#### 2.2.2 Strength Assessment According to the European Working Group on Sarcopenia in Older People

Handgrip strength (with Jamar dynamometer) and chair stand test were performed according to the EWOSP2 guidelines at age 63 [15].

#### 2.2.3 Aerobic Capacity

Aerobic capacity was estimated by a submaximal cycle test. At ages 27, 34, 52 and 63, all participants performed the Åstrand submaximal exercise test on a cycle ergometer [S10]. In short, the resistance (W) and the steady state heart rate were registered after 5–6 min of work. Absolute aerobic capacity (L·min^−1^) was estimated using an Åstrand nomogram and adjusted for age [S10]. The relative aerobic capacity was calculated (mL·kg^−1^·min^−1^). At age 16, a 9‐min run test was performed [S8, S9]. A subgroup of boys and girls also performed the Åstrand test [S10]. A regression equation for the conversion of the distance covered in the 9‐min run into relative aerobic capacity (mL·kg^−1^·min^−1^ and L·min^−1^) was created from this subgroup and applied to the whole study group at age 16. The absolute aerobic capacity (L·min^−1^) was thereafter calculated by multiplying the converted relative aerobic capacity (mL·kg^−1^·min^−1^) with body weight (kg) × 1000.

#### 2.2.4 Muscular Endurance

The muscular endurance of the arm and chest muscles was measured by the bench press test at ages 16, 34, 52 and 63. The number of lifts to straight arms at a rate of 25 per minute was counted in one trial [S1, S11]. Different weights were used for men (20 kg) and women (12 kg).

#### 2.2.5 Muscular Power

At age 16, 27, 34 and 63 the muscular power of the legs was measured by the Sargent jump test [S12]. Standing with the side to the wall, the inner arm was raised to mark how high the participant could reach. Thereafter, a countermovement jump with arm swing was performed with the hand touching the wall as high as possible. The distance between standing and jumping height was calculated (cm). Jump height can be considered an approximative measure of jump power per kg body weight [S13, S14].

#### 2.2.6 Lifestyle and Educational Background

Based on the replies from the question ‘What is you highest education?’ at ages 34 to 63, participants were classified as either having (1) or not having (0) a university degree. For the physical activity, at age 16 the question was: (1) Do you participate in leisure time sports activities? (yes/no). At the ages of 34 to 63, the question asked was: ‘Do you do any physical activity in your free time (including e.g. walking or gardening)?’ (yes/no). At the ages 34 to 63, the participants were asked the question ‘Do you smoke?’ (yes/no), and ‘How do you perceive your overall health’ (3‐grade scale) to describe the cohort (Figure 1).

### 2.3 Statistical Analysis

**FIGURE 2:**
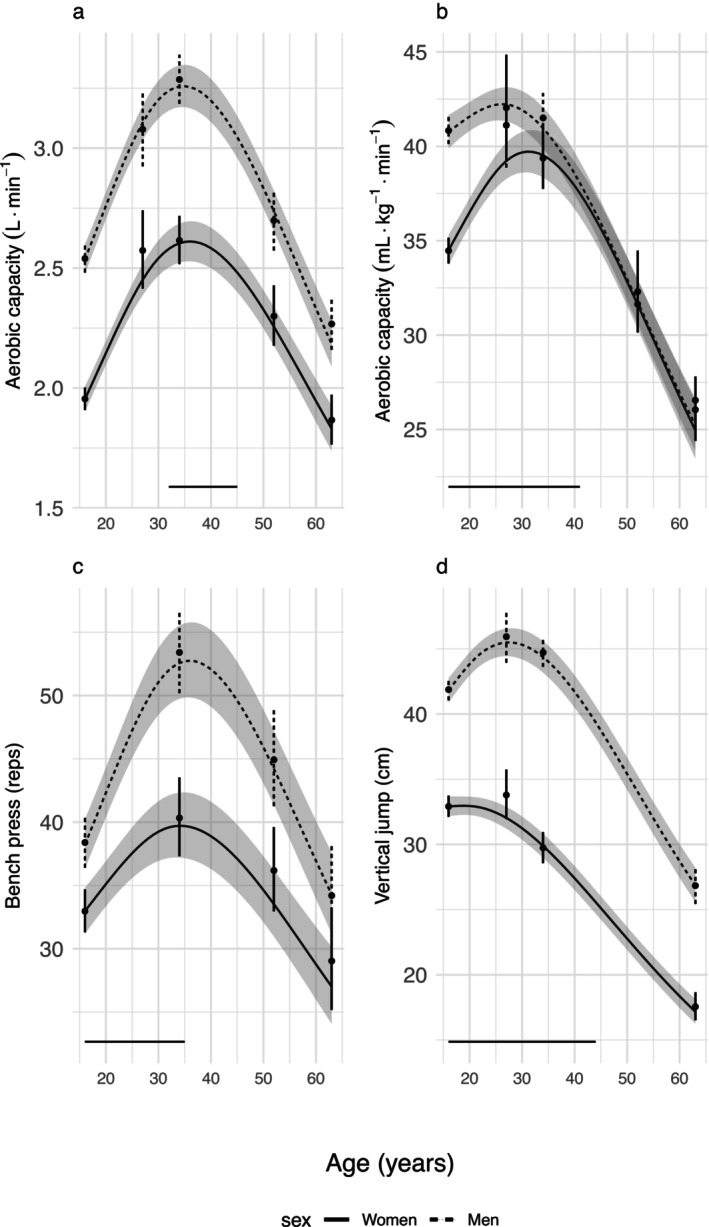
Physical capacity from 16 to 63 years of age. Line charts represent estimated values for the entire baseline cohort with 95% CI (shadowed area), back transformed to original scale. Dots represent observed values and SD. Lines on the *x*‐axis indicate a sex difference in slope in relative performance.

All statistical analyses were performed in R version 3.2 [S15]. A detailed description of the statistical analyses can be found in Data S1 (section Statistical analyses in the main paper). In brief, the data were analysed using linear mixed‐effects models using a statistical model including all original participants. Before statistical analysis, data were log transformed to make it conform to the linearFig model (Table 1). The effect of age and sex and the age‐by‐sex interaction were then tested for significance with models assuming age as a factor, estimating the mean at each time point when data were collected (factorial model) (Table 1). After that, more parsimonious models were explored that could make better predictions of performance outside of the measured time points, by fitting models with linear and curvilinear transforms of age (splines), optimising the knot location of the splines, moving the intercept from zero to the beginning of data collection and to the centred mean of age, fitting a random intercept with and without a random coefficient of age, and picking the model with the best fit to data (i.e., lowest Akaike information criterion; AIC) (optimised model) (Table 1). The best fitted models for each dependent variable were used to plot performance over the lifetime as a function of age, sex and age‐by‐sex (Table 2 and Figure 2 back transformed to original scale) (transformed variables in Figure S2). It was also used as a base model to test if lifestyle factors (university degree and physical activity) could add to the prediction of performance, over and above that of age and sex (Table 3 and Figures 5 and 6, back transformed to original scale) (transformed data in Figures S3 and S4). The intraclass correlation coefficient (ICC) was calculated from the random intercept model as the proportion of total error variance accounted for by the random intercept.

**TABLE 1:** Main effects, interactions, intra class coefficients (ICC) and variance for the physical capacity dependent variables.

	Aerobic capacity (L·min^−1^)	Aerobic capacity (mL·kg^−1^·min^−1^)	Bench press (reps)	Vertical jump (cm)
**Factorial model**				
Main effect sex: *p*‐value	< 0.001	0.007	< 0.001	< 0.001
Main effect age: *p*‐value	< 0.001	< 0.001	< 0.001	< 0.001
Age × Sex interaction: *p*‐value	0.025	< 0.001	0.018	< 0.001
ICC	0.35	0.29	0.50	0.40
**Optimised model**				
intercept_age	0	16	16	16
knot_age	29	27	31	21
main effect sex: *p*‐value	< 0.001	< 0.001	< 0.001	< 0.001
Main effect age: *p*‐value	< 0.001	< 0.001	< 0.001	< 0.001
Age × Sex interaction *p*‐value	0.027	< 0.001	0.003	< 0.001
**Predicted random effect variance**				
Variance at 16 years	0.003	0.001	0.036	0.007
Variance at 63 years	0.017	0.032	0.105	0.033
Variance increase	5.242	25.250	2.896	4.700
Chi square	52.64	151.05	81.60	105.31
*p*‐value	< 0.001	< 0.001	< 0.001	< 0.001

*Note:* Analysed by linear effect mixed models with age and sex in the models. To ensure consistency and simplify comparisons, a log transformation was applied to all datasets, as it effectively reduces skewness and approximates normality across varying distributions.

Abbreviation: Reps = number of repetitions.

**TABLE 2:** Estimated mean capacity, peak age and percentage change in physical capacity of all participants in the cohort.

	Age	16	20	25	30	35	40	45	50	55	60	63	Estimated peak capacity	Age at peak capacity (yrs)	% Decline from peak capacity to age 63
Aerobic capacity (L·min^**−**1^)	Men	2.54	2.76	3.02	3.20	3.26	3.20	3.05	2.83	2.59	2.33	2.18	3.26	35	33
	% change per year	N/A	2.06	1.61	0.85	0.04	−0.6	−1.18	−1.60	−1.92	−2.1	−2.2			
	Women	1.95	2.15	2.37	2.54	2.61	2.58	2.48	2.33	2.14	1.95	1.83	2.61	36	30
	% change per year	N/A	2.31	1.82	1.03	0.22	−0.5	−1.01	−1.44	−1.77	−2.0	−2.0			
Aerobic capacity (mL·kg^**−**1^·min^**−**1^)	Men	40.8	41.6	42.2	41.9	40.6	38.5	35.9	33.0	29.9	26.9	25.2	42.2	26	40
	% change per year	N/A	0.5	0.2	−0.3	−0.8	−1.2	−1.5	−1.8	−2.0	−2.1	−2.2			
	Women	34.5	36.5	38.6	39.7	39.4	37.9	35.6	32.8	29.8	26.7	24.9	39.7	31	37
	% change per year	N/A	1.4	0.9	0.2	−0.4	−1.0	−1.4	−1.8	−2.0	−2.2	−2.3			
Bench press (reps)	Men	38.2	42.3	47.2	51.1	52.7	52.1	49.6	45.9	41.5	36.9	34.3	52.7	36	35
	% change per year	N/A	2.5	2.0	1.2	0.3	−0.6	−1.2	−1.7	−2.1	−2.4	−2.5			
	Women	33.0	35.1	37.5	39.2	39.7	38.9	37.1	34.6	31.8	28.8	27.0	39.7	34	32
	% change per year	N/A	1.5	1.2	0.6	0.0	−0.6	−1.1	−1.5	−1.8	−2.0	−2.1			
Vertical jump (cm)	Men	41.8	43.8	45.3	45.3	44.0	41.7	38.7	35.4	32.0	28.7	26.9	45.5	27	41
	% change per year	N/A	1.1	0.4	−0.2	−0.8	−1.2	−1.6	−1.9	−2.1	−2.2	−2.2			
	Women	32.9	32.9	32.4	31.2	29.5	27.4	25.1	22.8	20.5	18.4	17.2	33.0	19	48
	% change per year	N/A	0.0	−0.5	−0.9	−1.3	−1.6	−1.8	−2.0	−2.1	−2.2	−2.2			

*Note:* The best fitted model for each dependent variable. The percentage change per year is calculated by comparison with the age 1‐year younger (e.g., ((50y‐49y)/49y)x100).

Abbreviation: Reps = number of repetitions.

**TABLE 3:** Association between physical capacity during life and educational level or physical activity respectively.

	Aerobic capacity (L·min^−1^)	Aerobic capacity (mL·kg^−1^·min^−1^)	Bench press (reps)	Vertical jump (cm)
**Being active at age 16 (yes/no)**				
Main effect of activity: *p*‐value	**< 0.001**	**< 0.001**	**< 0.001**	**0.019**
Interaction with age: *p*‐value	0.214	0.251	0.450	0.346
**Changing between active and inactive** ^a^				
Estimate (exp (b))^b^	1.07	1.06	1.11	1.04
95% confidence interval for exp. b	1.04–1.10	1.03–1.09	1.06–1.17	1.01–1.07
Main effect of changing: *p*‐value	**< 0.001**	**< 0.001**	**< 0.001**	**0.016**
**Having a university degree (yes/no)**				
Main effect of university degree: *p*‐value	**0.044**	0.711	**0.024**	0.201
Interaction with age: *p*‐value	0.281	0.365	0.492	0.837

Abbreviation: Reps = number of repetitions.

^a^ Adjusted for individual differences.

^b^ Since all data are log transformed, the b coefficient has been exponentiated to exp (b), so it represents the multiplicative effect on the dependent variable.

The log‐transformed variable property allows us to calculate and compare the percentage change between two ages and between the sexes on the original scale values. The percentage change per year after peak performance was calculated by dividing the difference between the older and younger age results by the younger age.

## 3 Results

Participants in the current study were representative of the original cohort in terms of sex, geographic region, body composition and adolescent muscular fitness at all visits (see ‘Representativity’ in Data S1 and Table S2). Factors indicating higher socioeconomic status (i.e., having a university degree) or being leisure‐time physically active at baseline increased the likelihood of participation in the study. At the age of 63 years, 33 participants (8%) were diseased, and 3% did not have a registered address and could not be invited (see Table S1). However, in the present study, the entire baseline cohort is included in the statistical modelling, minimising the potential bias due to dropouts.

BMI increased mainly between the ages of 16 and 50, with a faster increase in men between the ages of 30 and 50 (Figure 1a,b). At the age of 16 years, men were slightly more active in their leisure time, while women caught up by the age of 34 (Figure 1c). A detailed presentation of the observed anthropometric and physical capacity test results can be found in Table S3–S4. At the age of 63, no men, but four women [15] performed below the cut‐off value in either the handgrip test (< 16 kg) or the chair stand test (*n* < 10/30 s) according to EWGSOP2 [15] (Table S5).

### 3.1 Change in Performance With Age

For all physical capacity tests, the factorial models showed that there were differences in performance between the sexes, that performance changed with increasing age and that there was an interaction between age and sex (Table 1). The estimated means and 95% confidence intervals for physical capacity for the entire baseline cohort and for the ages between the observed measurements by the best fitting models are shown in Table 2 and Figure 2a–d. The estimated mean age of peak performance, relative change with age and group variance in performance at age 16 and 63 are presented in Table 2.

#### 3.1.1 Aerobic Capacity

Absolute aerobic capacity (L·min^−1^) was higher in men than in women throughout adult life (Figure 2a, Table 2). The relative increase to peak performance, age of peak performance (35 years in women and 36 years in men) and the rate of relative decline after age ~45 years was similar in men and women (Table 2 and Figure 2a). The rate of decline accelerated from 0.01 to 0.02 L·min^−1^ (~0.5%) per year at age 40 years to 0.04–0.05 L·min^−1^ (~2%) per year at age 63 years. The cumulative decline in absolute aerobic capacity from the age of peak capacity to 63 years of age was 30% and 33% in women and men respectively (Table 2). The ICC indicated that 35% of the error variance in the log transformed variable was due to stable individual differences over the study period. The group variance in performance increased fivefold from baseline to age 63 indicating that the difference between individuals in absolute aerobic capacity increased with age (Table 1).

Relative aerobic capacity (mL·kg^−1^·min^−1^) was higher in men than in women at younger ages, whereas there was no difference at older ages (Table 2 and Figure 2b). Up to peak performance, the women increased their relative aerobic capacity more than men. In parallel with the sex differences in changes in body weight and height (Figure 2b and Table S3), relative aerobic capacity peaked earlier in men (age 26) than in women (age 31). After peak performance, relative aerobic capacity gradually declined, and from the age of ~40, the relative decline was similar in both sexes (Table 2 and Figure 2b). The decline in relative aerobic capacity was ~0.4 mL·kg^−1^·min^−1^ (~1.1%) per year at age 40 years and increased to ~0.6 mL·kg^−1^·min^−1^ (~2.2%) per year at age 63 years. The cumulative decline in relative aerobic capacity from the age of peak capacity to age 63 was 37% in women and 40% in men (Table 2). The ICC indicated that 29% of the error variance on the log transformed variable was due to stable individual differences over the study period. The variance in relative aerobic capacity was 25 times higher in older than in younger ages, indicating that the difference between individuals in relative aerobic capacity increased with age (Table 1).

#### 3.1.2 Muscular Endurance

Men performed higher than women throughout life in the bench press test, even though women lifted a 40% lower weight than men. Up to peak performance, men increased their performance more than women. The age of peak performance (34 years in women and 36 years in men) and the rate of decline in performance after peak were similar in men and women (Table 2 and Figure 2c). The decline in muscular endurance was ~0.3 repetitions (0.6%) per year at age 40 and increased to ~0.7 repetitions (~2.3%) per year at age 63. The cumulative decline in performance from peak age to 63 years of age was 32 years for women and 35% for men (Table 2). The ICC indicated that 50% of the error variance in the log transformed variable was due to stable individual differences over the study period. The variance was threefold higher at older ages than at younger ages, indicating that the difference between individuals in muscular endurance increased with age (Table 1).

#### 3.1.3 Muscular Power

Jumping height was chosen as a measure of muscular power. The jumping height of men was higher than that of women from baseline to age 63 years. Up to peak performance, men (peak age 27 years) increased their performance more than women (peak age 19 years). After peak performance, jumping performance gradually declined and from the age of ~45 years, the relative decline was similar for both sexes (Table 2 and Figure 2d). At age 40, the annual decline in jump height was ~0.5 cm (1.2% and 1.6% for men and women, respectively). At the age of 63, the annual decline was still ~0.5 cm, corresponding to a 2.2% yearly decline. From peak age to age 63 years, the cumulative decline in jump height was 48% during 44 years in women and 41% during 36 years in men (Table 2). The ICC indicated that 40% of the error variance in the log transformed variable was due to stable individual differences over the study period. The variance in jump height was almost 5 times as high at older ages than at younger ages, indicating that the difference between individuals in jumping height increased with age.

### 3.2 Individual Trajectories and Association With Educational Level and Physical Activity

**FIGURE 3:**
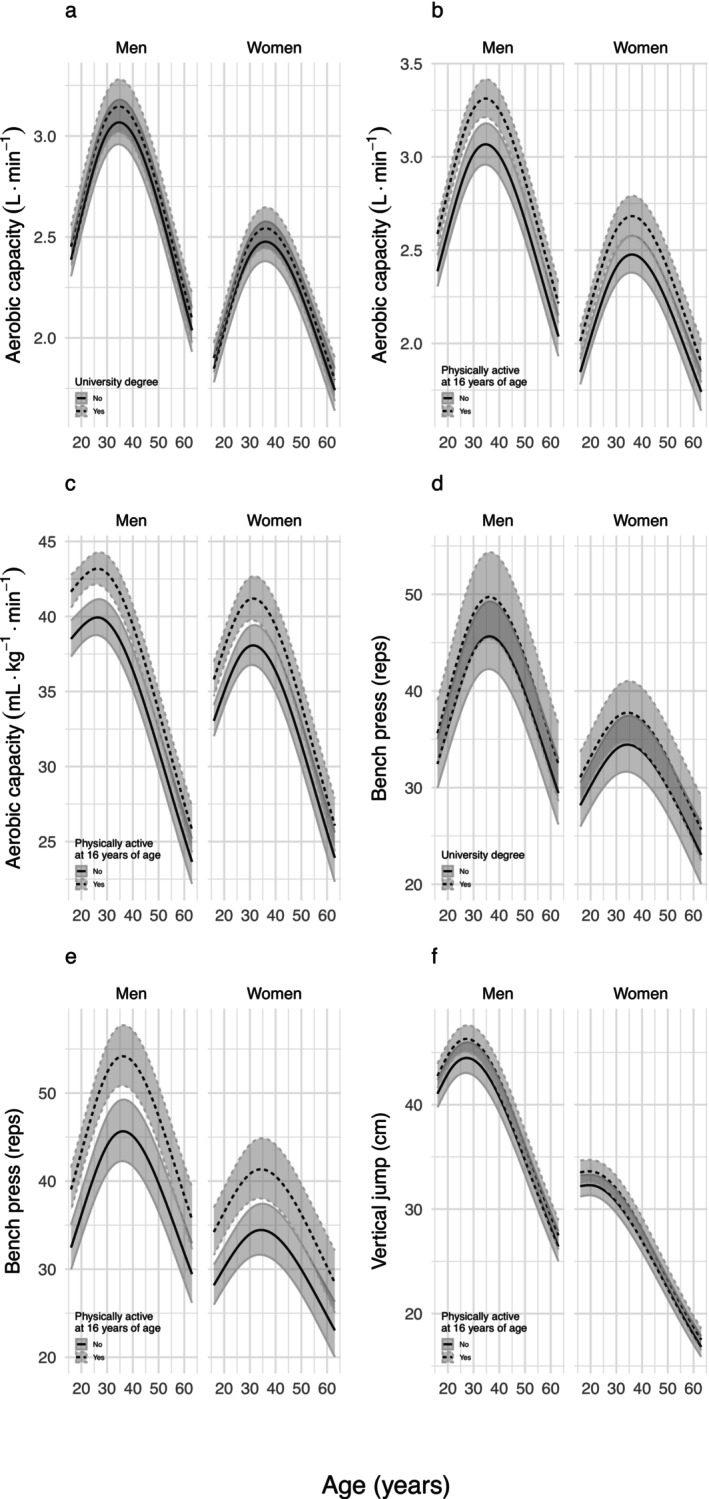
Effect on physical capacity of being physically active at age 16 or having a university degree at any age. Only capacities with a statistically significant association with physical activity or education are shown. Line charts represent estimated values for the entire baseline cohort with 95% confidence intervals (shaded area), back‐transformed to the original scale.

**FIGURE 4:**
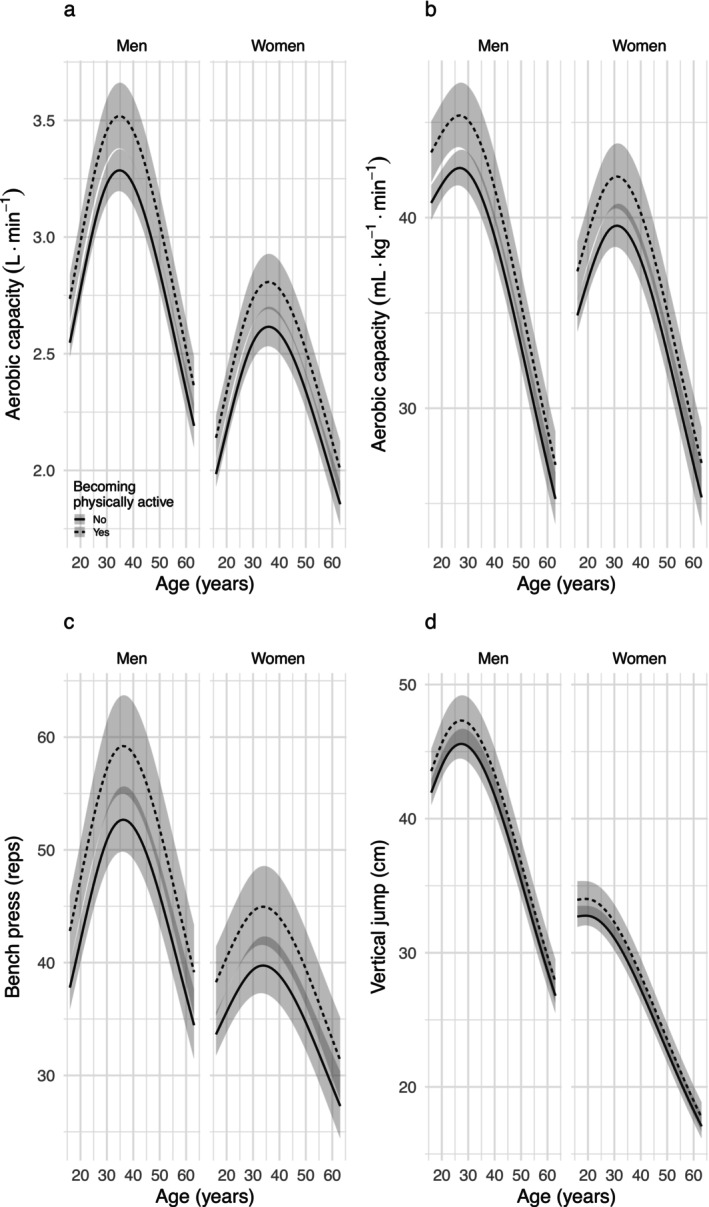
Effect on physical capacity of changing between physically active and inactive during life. Line charts represent estimated values for the entire baseline cohort with 95% CI (shadowed area), back transformed to original scale.

Participants who were physically active in their leisure time at the age of 16 years had higher aerobic capacity (both relative and absolute), muscular endurance and muscular power in adulthood than inactive participants (Table 3, Figure 3). Switching from a physically inactive to an active lifestyle also increased physical capacity in all these tests by 6%–7% for aerobic capacity, 11% for the bench press and 4% for the vertical jump (Table 3, Figure 4). The significant main effects also showed that those who had a university degree had higher absolute aerobic capacity and muscular endurance compared to those who did not have a university degree (Table 3, Figure 3). There were no interactions with age, indicating that the effect of being leisure‐time physically active at age 16 or having a university degree at any age on physical capacity remained significant at all ages during the observation period.

## 4 Discussion

In this study, we report longitudinal changes in physical capacity from adolescence to early old age (16 to 63 years) using repeated objective measures of aerobic capacity and, muscular endurance and power in a population‐based Swedish cohort born in 1958 [14]. The main finding was that peak capacity was reached before the age of 36 years and that after the age of ~40 years there was a similar and accelerating decrease in all the tested capacities in both sexes. After the peak, the annual decrease accelerated from an average of < 1% per year in the first decade to > 2.0% in the last decade of the observation period. On average, the loss of physical capacity in both sexes was 37% from peak age to age 63, ranging from 30% to 48%.

With regards to the age at which peak capacity is reached and the progressive, non‐linear decline after peak capacity, our data are consistent with longitudinal data from master athletes [6, 16, 17]. However, both the cumulative loss from peak capacity to age 63 and the annual decline in capacity are larger than the values reported in master athletes. At the age of 63, the women and men maintained only ~65% of their peak capacity in aerobic capacity and muscular endurance. This compares to athletes of the same age who maintained > 80% of their peak capacity in endurance activities [8, 9, 10]. Similarly, for muscular power, women in the present study maintained only half and men 60% of their peak muscular power at age 63, a decline that master athletes do not reach until > 75 years of age [7, 8, 9, 10]. The advantage of the comparison with athletes is that their decline is less influenced by physical inactivity and that the decline may reflect an inherent biological aging process. Age‐related changes in the neuromuscular system are progressive and suggest mitochondrial dysfunction as an early driver of performance decline in the 30s, with remodelling of the extracellular matrix and neuromuscular junctions and later atrophy (catabolism), anabolic resistance, inflammatory and proliferative changes exacerbating the loss of mass and strength [18, 19, 20]. These changes also apply to trained athletes (30). Shortly after peak performance, however, the signs are likely to be quite discrete, showing a reduced adaptation to exercise, perhaps due to a decline in cell respiratory capacity, the quality of the extracellular matrix, a remodelling and small reduction in the number of motor units. Future studies should investigate the early phase of physical performance decline to understand what drives the decline and provide evidence for rational intervention.

However, one question that has not yet been answered is whether the extent of the decline in physical capacity with increasing age observed in cross‐sectional studies in the general population with the known limitations can be confirmed in longitudinal studies. In our study, we had the unique opportunity to investigate the change in physical capacity longitudinally over a period of 47 years. Compared to our study and similar to the results of studies combining cross‐sectional with short‐term longitudinal data [16, 21, 22], the decline in physical capacity from cross‐sectional studies appears to be underestimated. Regarding the sex difference in the rate of decline in physical capacity, cross sectional studies have not been able to establish whether the rate of decline in women compared to men occur at a similar [23, 24, 25, 26, 27, 28], faster [12, 29] or slower [11, 28, 30, 31] rate. We followed the same individuals for 47 years and, consistent with findings from elite athletes [6, 16], we found that there is no sex difference in decline with age. In summary, our data suggest that the general shape of the performance curve in adulthood is similar in men and women and that this shape is innate rather than acquired through lifestyle. However, the slope of the increase before, and the decrease after the peak, as well as the overall level of performance, likely depends on the extent to which an individual realises their full adaptive potential by being physically active at each age of adulthood.

Physical activity consistently emerges as the most important factor influencing both absolute physical capacity and the rate of age‐related decline. Our longitudinal data are consistent with previous studies showing that regular physical activity can attenuate the decline in physical performance [17, 32, 33, 34, 35, 36, 37]. Individuals who were physically active in their leisure time at age 16 maintained higher aerobic capacity, muscular endurance and muscle power throughout the observation period. This emphasises the importance of early intervention to establish positive exercise habits in adolescence and early adulthood, as these patterns appear to have long‐term benefits for physical function.

Encouragingly, our results show that transitioning from physical inactivity to activity at any age significantly improves performance in all fitness modalities studied. These findings contradict the assumption that early inactivity irreversibly impairs physical performance. Rather, taking up regular physical activity leads to measurable improvements in performance even in later decades of life. This finding is of particular importance for clinical practice, as physical activity is still the only evidence‐based intervention to reduce the risk of sarcopenia [2, 38]. Recent large population studies also show that an active lifestyle is beneficial at any age [13, 39, 40].

Educational level proved to be another important predictor of physical performance in our study. Individuals with a university degree had higher absolute aerobic capacity and muscular endurance, but not higher muscular power, than people without a university degree. These finding warrants further investigation, particularly given the clinical significance of studies linking lower educational attainment to increased risk of sarcopenia [41, 42, 43].

The ICC indicated that stable individual differences across the lifespan accounted for 30%–50% of the error variance in performance. This indicates that individuals that start out as high performers are likely to continue to perform well during their adulthood. This is worth recognising in view of the secular development of physical capacity. Adolescents born 20 years later than the SPAF‐1958 cohort were found to have lower aerobic and muscular upper body capacity compared to the SPAF‐1958 cohort at the same age [44]. This trend suggests that future generations may start with lower peak physical capacity and face muscle weakness earlier in life. With increasing age, the difference between the weakest and strongest performers widened: from 16 to 63 years, the inter‐individual variances in the capacities studied increased 3‐ to 25‐fold. This important finding underpins the concept that every person realises their physical potential to a different extent over the course of their life. The considerable inter‐individual differences in physical decline suggest that both internal physiological factors and external environmental conditions significantly influence the maintenance of a person's physical performance over time. These differences point to modifiable factors that should be considered in interventions to optimise physical function throughout adulthood.

Despite the protective effects of physical activity and higher education, our data suggest that no one can escape age‐related decline in physical capacity. Interestingly, we found that physical performance begins to decline around the age of 35, significantly earlier than many age‐related changes commonly cited as explanations for reduced capacity.

Age‐related neuromuscular changes—such as reduced innervation of motor units, a shift towards slow‐twitch fibres, muscle fibre atrophy and increasing fibrosis of muscle and connective tissue—generally do not become apparent until later decades [2, 4]. Although physical activity influences many of these factors, our results suggest that the age at which peak performance is reached cannot be significantly influenced by external factors such as the level of physical activity. However, the rate of later decline appears to be modifiable by physical activity, which is consistent with the established literature on the importance of remaining active during aging (idem, reviewed in [2, 38]).

## 5 Strengths and Limitations

The longitudinal design of our study offers several methodological advantages over cross‐sectional studies. Our statistical model includes all original participants, addressing the common issue of systematic dropouts in longitudinal research and allowing us to predict the decline in physical capacity for the entire initial cohort. This approach significantly reduces the selection bias that usually favours the healthier participants at follow‐up. The comprehensive test battery measured three different areas of physical fitness—aerobic capacity, muscular endurance and strength/power—providing a multidimensional view of physical function that is rarely captured in studies of aging. In addition, the 47‐year follow‐up period to age 63 provides unique insights into the early functional decline which may develop into clinically significant sarcopenia.

There are also limitations that should be noted. First, although the measures of physical capacity are comprehensive, they may not capture all relevant aspects of physical function that are important in old age. Second, we used a simple yes/no question about leisure‐time physical activity across ages 16 to 63 to ensure consistency and reduce measurement error. Despite its simplicity, this question showed a significant association with physical performance, suggesting it has meaningful validity. We lack detailed objective information on the intensity, duration and consistency of physical activity across the lifespan, which may influence the observed associations. More detailed or objective data on physical activity would make it possible to present differentiated activity groups and thus further emphasize the importance of physical activity. Objective measures of physical activity should be used in future studies to learn more about the longitudinal effects of activity on the decline in physical capacity with age.

Finally, our cohort represents a Swedish population born in 1958, which may limit generalisability to other birth cohorts or cultural contexts with different physical activity patterns, educational opportunities or health care systems.

## 6 Conclusion

This 47‐year longitudinal study of physical capacity from adolescence (age 16) to early old age (age 63) in a population‐wide Swedish cohort reveals several important patterns. Physical capacity follows a constitutive trajectory with modality‐ and sex‐specific peaks between 19 and 36 years of age, followed by a steeper decline than previously reported in cross‐sectional studies. This accelerated decline represents the actual longitudinal change and not the attenuated slopes typically observed in cross‐sectional studies. The early onset of decline—before the age of 40—presents a challenge to existing strategies to prevent its progression into clinically overt sarcopenia. While the timing of peak capacity appears to be largely invariant, our results show that three key aspects of physical capacity are modifiable: the rate of improvement before peak performance, the absolute level of performance achieved, and the rate of decline after peak performance. As all three aspects can be positively influenced by physical activity, they have important clinical and public health implications.

## Ethics Statement

The study protocol was in accordance with the Declaration of Helsinki and was approved by the Umeå University Human Research Ethics Committee (Dnr 09‐082M), and the Swedish Ethical Review Authority (Dnr 2020‐04338 and Dnr 2023‐04163‐0). Participation was voluntary, and all participants signed an informed consent form. All authors of this manuscript comply with the guidelines of ethical authorship and publishing in the *Journal of Cachexia, Sarcopenia and Muscle* [S16].

## Conflicts of Interest

The authors declare no conflicts of interest.

## Supporting information


**Figure S1:** Flow chart showing the part of the population participating at baseline and follow‐ups.

**Table S1:** Age, number of and cause of death in the cohort.

**Table S2:** Participating in testing at the third follow‐up (age 63). Significant factors from the logistic regression, estimating the odds ratio (OR) using baseline variables (age16), presented for each group of variables.

**Table S3:** Observed values for anthropometric measurements.

**Table S4:** Observed values for physical capacity measurements at the ages of follow‐up.

**Table S5:** Observed values for functional capacity at age 63.

**Data S1:** Supplementary Information.

**Data S2:** Supplementary Information.
