# Supplementary figures and images for: Rise and Fall of Physical Capacity in a General Population: A 47‐Year Longitudinal Study
Source: J Cachexia Sarcopenia Muscle. 2025 Nov 16;16(6):e70134. doi: 10.1002/jcsm.70134 (PMC12620399; doi:10.1002/jcsm.70134)

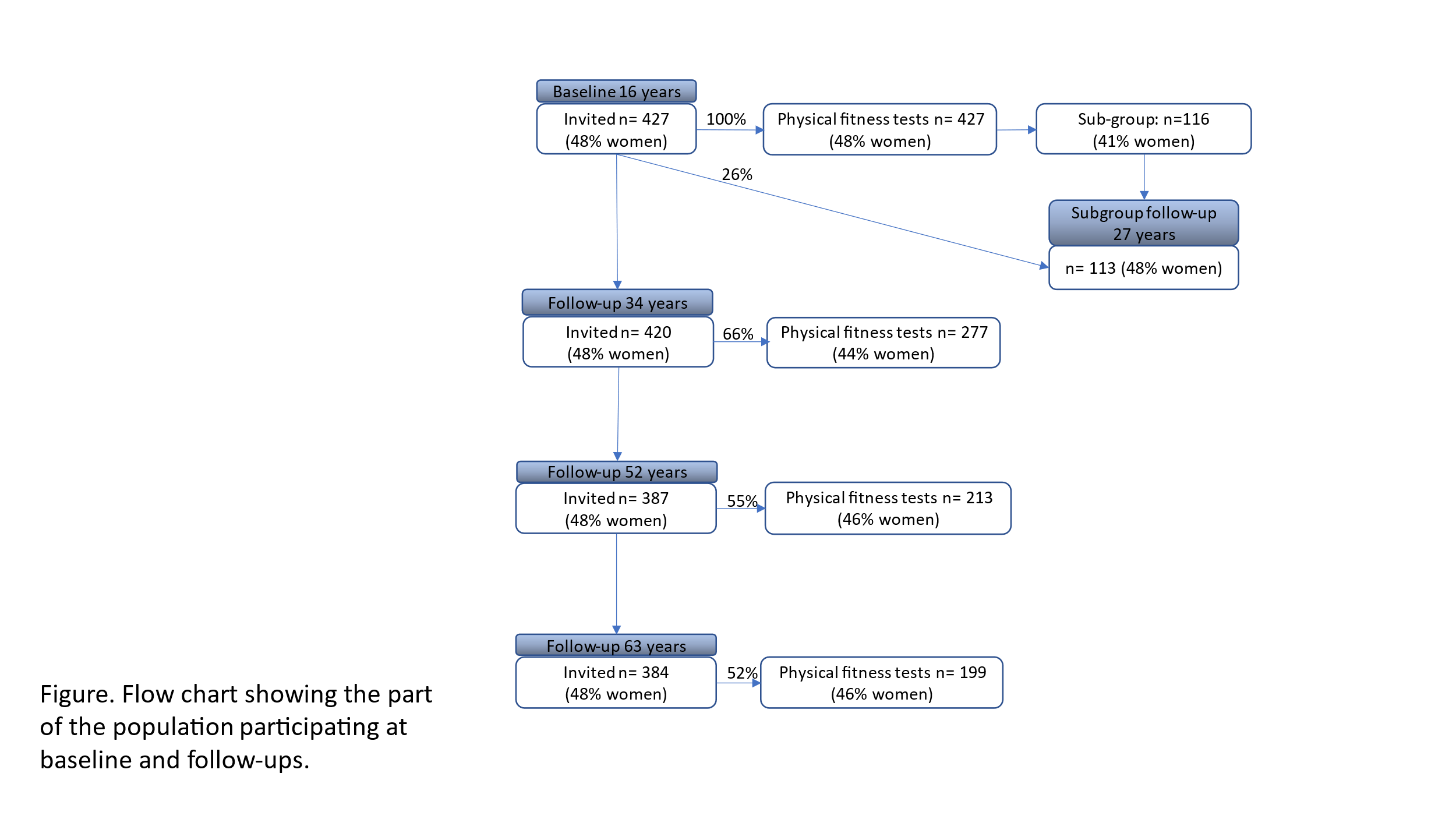

Supplement: Supplementary file 1 — Figure S1: Flow chart showing the part of the population participating at baseline and follow‐ups. [file JCSM-16-e70134-s003.docx]
