# Supplementary material for: Rise and Fall of Physical Capacity in a General Population: A 47‐Year Longitudinal Study
Source: J Cachexia Sarcopenia Muscle. 2025 Nov 16;16(6):e70134. doi: 10.1002/jcsm.70134 (PMC12620399; doi:10.1002/jcsm.70134)
Supplement: Supplementary file 2 — Table S1: Age, number of and cause of death in the cohort. [file JCSM-16-e70134-s001.docx]

**Table S1.** Age, number of, and cause of death in the cohort

| **Number (%)** | | | | | |
| --- | --- | --- | --- | --- | --- |
| **Age at death** | **Total**  **M/W** | **Cancer** | **CVD** | **Alcohol related** | **Unknown/Other** |
| 20-30y | 2/0 |  |  |  | 2 |
| 30-39y | 1/1 |  |  |  | 2 |
| 40-49y | 3/0 |  | 2 | 1 |  |
| 50-59y | 11/5 | 7 (44%) | 4 (25%) | 4 (25%) | 1 (6%) |
| 60-64y | 6/4 | 6 (55%) | 2 (18%) | 2 (18%) | 1 (9%) |

M = men, W = women, CVD = cardiovascular disease
