# Supplementary material for: Rise and Fall of Physical Capacity in a General Population: A 47‐Year Longitudinal Study
Source: J Cachexia Sarcopenia Muscle. 2025 Nov 16;16(6):e70134. doi: 10.1002/jcsm.70134 (PMC12620399; doi:10.1002/jcsm.70134)
Supplement: Supplementary file 3 — Table S2: Participating in testing at the third follow‐up (age 63). Significant factors from the logistic regression, estimating the odds ratio (OR) using baseline variables (age16), presented for each group of variables. [file JCSM-16-e70134-s004.docx]

**Table S2.** Participating in testing at the third follow-up (age 63). Significant factors from the logistic regression, estimating the odds ratio (OR) using baseline variables (age16), presented for each group of variables

| **Variables at 16 years of age** | | | | |  | **Distribution of characteristic** | | |
| --- | --- | --- | --- | --- | --- | --- | --- | --- |
| **Group** | **Significant factor in the group** | **OR** | **95% CI** | **P-value** |  | **Group** | **Participants** | **Non- participants** |
| Socio-demographic characteristics | Upper secondary school program (P=0,T=1) | 1.78 | 1.19-2.66 | <0.01 |  | T (%) | 42 | 30 |
| Body dimensions | None | | | |  |  |  |  |
| Physical fitness | None | | | |  |  |  |  |
| Physical activity | Participated in leisure time sports activity (no=0, yes=1) | 1.88 | 1.23-2.87 | <0.01 |  | Yes (%) | 68 | 53 |
| Attitudes to physical education | Felt satisfied with their performance at physical education (1-5) | 1.45 | 1.13-1.87 | <0.01 |  | Almost always /Always (%) | 49 | 36 |

No interaction between sex and any of the variables

For information about the variables that are included in each group, see Participants and drop-out.

CI= confidence interval of OR

P=practical, T= theoretical

Satisfied with performance at physical education: 1 (never) 5 (always)
