# Supplementary material for: Rise and Fall of Physical Capacity in a General Population: A 47‐Year Longitudinal Study
Source: J Cachexia Sarcopenia Muscle. 2025 Nov 16;16(6):e70134. doi: 10.1002/jcsm.70134 (PMC12620399; doi:10.1002/jcsm.70134)
Supplement: Supplementary file 4 — Table S3: Observed values for anthropometric measurements. [file JCSM-16-e70134-s008.docx]

**Table S3.** Observed values for anthropometric measurements

|  |  |  |  | **Men** |  |  |  |  |  |  | **Women** |  |  |  |
| --- | --- | --- | --- | --- | --- | --- | --- | --- | --- | --- | --- | --- | --- | --- |
| **Age** | **N** | **Minimum** | **25th percentile** | **Median** | **75th percentile** | **Maximum** |  | **N** | **Minimum** | **25th percentile** | **Median** | **75th percentile** | **Maximum** |  |
|  | **Height (cm)** | |  |  |  |  |  |  |  |  |  |  |  |  |
| **16** | 222 | 144.0 | 172.0 | 175.0 | 180.0 | 192.0 |  | 205 | 148.0 | 162.0 | 167.0 | 170.0 | 186.0 |  |
| **27** | 64 | 164.0 | 175.0 | 179.0 | 184.3 | 192.0 |  | 43 | 158.0 | 165.0 | 170.0 | 171.5 | 176.0 |  |
| **34** | 157 | 162.0 | 176.0 | 180.0 | 184.6 | 197.2 |  | 121 | 155.3 | 163.5 | 168.6 | 171.4 | 187.0 |  |
| **52** | 112 | 161.3 | 176.2 | 180.5 | 184.5 | 198.2 |  | 98 | 150.3 | 163.2 | 168.5 | 171.3 | 187.5 |  |
| **63** | 109 | 167.0 | 176.0 | 180.0 | 184.0 | 196.7 |  | 91 | 152.1 | 162.9 | 168.0 | 170.9 | 186.5 |  |
|  | **Body weight (kg)** | | |  |  |  |  |  |  |  |  |  |  |  |
| **16** | 222 | 41.4 | 56.9 | 62.0 | 68.0 | 96.0 |  | 205 | 41.3 | 52.0 | 55.7 | 61.0 | 97.5 |  |
| **27** | 64 | 56.0 | 68.0 | 74.5 | 80.3 | 102.0 |  | 43 | 51.0 | 56.5 | 60.0 | 65.0 | 78.0 |  |
| **34** | 157 | 55.2 | 73.0 | 79.8 | 84.8 | 110.6 |  | 121 | 50.8 | 59.8 | 64.4 | 72.8 | 135.8 |  |
| **52** | 113 | 56.4 | 77.6 | 84.3 | 93.4 | 141.2 |  | 98 | 51.3 | 63.6 | 68.4 | 77.8 | 125.6 |  |
| **63** | 109 | 60.3 | 77.0 | 84.5 | 94.6 | 127.2 |  | 91 | 53.0 | 64.5 | 71.0 | 77.5 | 109.4 |  |
|  | **Body mass index (kg**·**m^-2^)** | | |  |  |  |  |  |  |  |  |  |  |  |
| **16** | 222 | 14.8 | 18.7 | 20.0 | 21.3 | 33.8 |  | 205 | 16.1 | 18.9 | 20.2 | 21.9 | 31.5 |  |
| **27** | 64 | 18.9 | 21.5 | 22.8 | 24.1 | 33.3 |  | 42 | 18.4 | 20.4 | 21.6 | 22.7 | 27.5 |  |
| **34** | 157 | 19.1 | 22.7 | 24.1 | 26.0 | 33.5 |  | 121 | 18.6 | 21.3 | 22.9 | 25.2 | 43.1 |  |
| **52** | 112 | 19.5 | 24.6 | 26.6 | 28.3 | 42.2 |  | 98 | 19.2 | 22.7 | 25.1 | 27.0 | 43.7 |  |
| **63** | 109 | 19.2 | 24.4 | 26.5 | 28.3 | 36.8 |  | 91 | 18.6 | 23.5 | 25.2 | 27.5 | 38.0 |  |
|  | **Waist circumference (cm)** | | | |  |  |  |  |  |  |  |  |  |  |
| **34** | 156 | 72.7 | 85.1 | 88.8 | 94.6 | 115.5 |  | 119 | 63.9 | 73.8 | 77.7 | 83.6 | 131.1 |  |
| **52** | 113 | 78.0 | 90.5 | 98.5 | 104.0 | 136.5 |  | 98 | 66.0 | 80.6 | 85.6 | 93.0 | 130.0 |  |
| **63** | 106 | 82.0 | 93.0 | 99.0 | 106.0 | 123.5 |  | 91 | 73.0 | 83.8 | 90.0 | 96.0 | 115.0 |  |
|  |  |  |  |  |  |  |  |  |  |  |  |  |  |  |
