# Supplementary material for: Rise and Fall of Physical Capacity in a General Population: A 47‐Year Longitudinal Study
Source: J Cachexia Sarcopenia Muscle. 2025 Nov 16;16(6):e70134. doi: 10.1002/jcsm.70134 (PMC12620399; doi:10.1002/jcsm.70134)
Supplement: Supplementary file 5 — Table S4: Observed values for physical capacity measurements at the ages of follow‐up. [file JCSM-16-e70134-s005.docx]

**Table S4.** Observed values for physical capacity measurements at the ages of follow-up

|  |  |  |  | **Men** |  |  |  |  |  |  | **Women** |  |  |  |
| --- | --- | --- | --- | --- | --- | --- | --- | --- | --- | --- | --- | --- | --- | --- |
| **Age** | **N** | **Minimum** | **25th percentile** | **Median** | **75th percentile** | **Maximum** |  | **N** | **Minimum** | **25th percentile** | **Median** | **75th percentile** | **Maximum** |  |
|  | **Aerobic capacity (L**·**min^-^1)** | | | | |  |  |  |  |  |  |  |  |  |
| **16** | 214 | 1.5 | 2.3 | 2.6 | 2.8 | 3.9 |  | 197 | 1.1 | 1.8 | 1.9 | 2.1 | 3.3 |  |
| **27** | 52 | 1.7 | 2.7 | 3.1 | 3.4 | 4.2 |  | 32 | 1.5 | 2.3 | 2.6 | 2.9 | 3.5 |  |
| **34** | 157 | 2.0 | 2.8 | 3.3 | 3.7 | 6.6 |  | 121 | 1.6 | 2.2 | 2.5 | 3.0 | 4.6 |  |
| **52** | 100 | 1.5 | 2.3 | 2.8 | 3.1 | 4.6 |  | 88 | 1.0 | 1.9 | 2.3 | 2.8 | 3.7 |  |
| **63** | 103 | 1.3 | 1.9 | 2.3 | 2.6 | 4.3 |  | 81 | 1.0 | 1.6 | 1.8 | 2.2 | 3.5 |  |
|  | **Aerobic capacity (mL**·**kg^-^1**·**min^-^1)** | | |  |  |  |  |  |  |  |  |  |  |  |
| **16** | 216 | 26.8 | 37.5 | 41.5 | 44.4 | 57.4 |  | 197 | 20.4 | 32.7 | 34.4 | 36.7 | 56.9 |  |
| **27** | 52 | 28.6 | 34.5 | 40.2 | 47.3 | 62.7 |  | 32 | 27.8 | 37.6 | 43.8 | 46.8 | 56.5 |  |
| **34** | 157 | 24.0 | 36.0 | 42.0 | 48.0 | 85.0 |  | 121 | 20.0 | 33.0 | 39.0 | 46.0 | 74.0 |  |
| **52** | 100 | 18.9 | 25.9 | 31.7 | 37.5 | 55.8 |  | 88 | 12.7 | 27.0 | 32.5 | 38.1 | 61.4 |  |
| **63** | 103 | 11.9 | 22.6 | 26.1 | 32.5 | 50.7 |  | 81 | 13.6 | 21.6 | 26.2 | 31.6 | 45.3 |  |
|  | **Bench press (reps)** | | | |  |  |  |  |  |  |  |  |  |  |
| **16** | 213 | 4 | 30 | 39 | 50 | 100 |  | 201 | 7 | 26 | 32 | 40 | 100 |  |
| **34** | 157 | 21 | 40 | 52 | 70 | 187 |  | 118 | 9 | 30 | 40 | 53 | 106 |  |
| **52** | 105 | 6 | 35 | 43 | 56 | 114 |  | 93 | 7 | 27 | 36 | 48 | 142 |  |
| **63** | 104 | 0 | 27 | 35 | 46 | 119 |  | 84 | 0 | 19 | 27 | 35 | 203 |  |
|  | **Vertical jump (cm)** | | | |  |  |  |  |  |  |  |  |  |  |
| **16** | 215 | 24 | 39 | 42 | 46 | 60 |  | 202 | 16 | 29 | 34 | 37 | 49 |  |
| **27** | 57 | 20 | 42 | 47 | 51 | 65 |  | 29 | 23 | 30 | 34 | 38 | 46 |  |
| **34** | 155 | 27 | 40 | 45 | 50 | 61 |  | 119 | 13 | 26 | 30 | 34 | 50 |  |
| **63** | 104 | 9 | 24 | 28 | 32 | 43 |  | 85 | 8 | 15 | 18 | 21 | 28 |  |
|  |  |  |  |  |  |  |  |  |  |  |  |  |  |  |

N = number of participants that have conducted the test.
