# Supplementary material for: Rise and Fall of Physical Capacity in a General Population: A 47‐Year Longitudinal Study
Source: J Cachexia Sarcopenia Muscle. 2025 Nov 16;16(6):e70134. doi: 10.1002/jcsm.70134 (PMC12620399; doi:10.1002/jcsm.70134)
Supplement: Supplementary file 6 — Table S5: Observed values for functional capacity at age 63. [file JCSM-16-e70134-s002.docx]

**Table S5.** Observed values for functional capacity at age 63

|  |  |  |  | **Men 63 years** |  |  |  |  |  |  | **Women 63 years** |  |  |  |
| --- | --- | --- | --- | --- | --- | --- | --- | --- | --- | --- | --- | --- | --- | --- |
| **Test** | **N** | **Minimum** | **25th percentile** | **Median** | **75th percentile** | **Maximum** |  | **N** | **Minimum** | **25th percentile** | **Median** | **75th percentile** | **Maximum** |  |
| **Handgrip** | 108 | 32 | 43 | 48 | 54 | 86 |  | 90 | 14 | 24 | 28 | 31 | 48 |  |
| **Chair stand test** | 107 | 10 | 14 | 16 | 18 | 30 |  | 90 | 7 | 14 | 16.5 | 19 | 35 |  |
|  |  |  |  |  |  |  |  |  |  |  |  |  |  |  |

N = number of participants that have conducted the test.

The handgrip test was measured in kilograms and the chair stand test was measured in the number of chair stands in 30 seconds.
