# Supplementary material for: Rise and Fall of Physical Capacity in a General Population: A 47‐Year Longitudinal Study
Source: J Cachexia Sarcopenia Muscle. 2025 Nov 16;16(6):e70134. doi: 10.1002/jcsm.70134 (PMC12620399; doi:10.1002/jcsm.70134)
Supplement: Supplementary file 7 — Data S1: Supplementary Information. [file JCSM-16-e70134-s006.docx]

# Supplementary Material

## Participants and drop-out

At the beginning of the study, in 1974, almost 86000 pupils were enrolled in Swedish upper secondary high school. Two-thirds of all adolescents in 1974 went on to upper secondary school. A group of 427 (0.5%, 222 males, and 205 females) of these pupils was systematically selected: The country was stratified into six regions with a similar population size and climate. In each region, one or more secondary high schools were randomly selected. At each school, the number of pupils was selected in proportion to the distribution of girls and boys in secondary high school, the number of pupils in the region, and the proportion of pupils in each school program (theoretical three-year program to prepare for higher studies or practical two-year program to prepare for working life). Individual participants were then randomly selected at each school based on these criteria and are representative of 16-year-old girls and boys attending secondary high school in Sweden. The sampling procedure and the results from the baseline data collection have been described earlier [1,2].

From the selected group of 427 pupils, a subgroup of 116 participants (69 boys and 47 girls) consented to the donation of a piece of muscle through a muscle biopsy [2,3]. Eleven years later, at age 27, this subgroup was invited to a follow-up [4] (Figure S1).

The first follow-up of the entire study population was performed in 1992 when the participants were at age 34. All 420 participants who were alive and registered in Sweden were invited [5]. In 2010, at age 52, all 423 participants who were alive and registered in Sweden were invited [6]. Twenty of these participants lived far away from the testing sites and were only invited to answer the questionnaire. At age 63, all 384 participants who were alive and registered in Sweden were invited for testing and questionnaires (Figure S1). The number and causes of death are presented in Table S1. Fifteen of the participants took part only at baseline, twelve were involved in the study until the age of 34 and six until the age of 52 due to deaths before age of 63.

Longitudinal studies need to consider the possibility of bias due to non-random dropout of participants. A dropout analysis was therefore performed after each follow-up [4,6,7]. Ongoing study participants at all follow-ups were still representative of the original study cohort in terms of sex, adolescent geographical area, adolescent body composition and muscular fitness. However, at age 34, a higher average school grade at age 16 was associated with fewer dropouts at age 34 for boys, but not for girls [7]. Dropout at age 52 was related to aerobic capacity (for women) or sports club membership (for men) at age 16 [6]. At age 63, being physically active as an adolescent and attending a theoretical program at secondary high school decreased the risk for dropout.

At the age of 63, the dropout analyses were carried out in the same way as at the age of 52 [6]. In short, logistic regression was used to test which factors at baseline (16 years of age), were associated with higher odds for dropping out from answering the questionnaire as well as for dropping out from testing at age 63. A bivariate logistic regression was performed, and dropout (yes = 1, no = 0) at age 63 was used as the dependent variable. Independent variables assessed at baseline (16 years of age) were grouped according to type of variable. Each group of variables was analysed using the forward stepwise method (Wald) of logistic regression. The variables that were statistically significant in each group were combined in a final model. The following groups were used: Socio-demographics, Body dimensions, Performance in physical capacity tests, Physical activity and Attitudes to physical education [6]. The odds for participating in testing at age 63 of age was independent of sex and of body dimensions or physical test performance at age 16 (Table S2). For both men and women, the odds for participating in testing at age 63 was higher among those who attended theoretical compared to practical programs at secondary high school, were leisure-time sports active and felt satisfied with their performance during physical education at age 16. After entering all significant factors from the grouped analyses into one model, all factors still increased the odds for participating in testing at age 63: attending a theoretical program (OR=1.76, 95% CI=1.14-2.72, p<0.05) being leisure-time sports active (OR=1.61, 95% CI=1.03-2.51, p<0.05) and feeling satisfied with their performance during physical education at age 16 (OR=1.34, 95% CI for OR=1.03-1.74, p<0.05) at age 16. (Final model: p<0.001).

## Representativity

At baseline, the SPAF cohort was representative of the 75% of Swedish men and women who attended secondary high school in the 1970s. Consequently, more participants in the SPAF cohort had a university education (M:39%, W:54%) compared to the general population according to Statistics Sweden (M:32%, W:40%) [8]. In addition, the number of foreign-born participants in the SPAF 1958 cohort is probably lower than in the general population of this age group in Sweden in 2021. When the study began, 7% of the Swedish population was born abroad. Due to increasing immigration, this proportion rose to 20% in 2019 [9]. This is a factor that could also explain the differences seen in terms of education level compared to the Swedish population in general, as people with a foreign background tend to have a lower education level in general [8].

In the SPAF cohort, 8% died between the ages of 16 and 63. This is slightly lower than the mortality rate of 11% in the total population born in Sweden in 1958 [10], which includes people who immigrated after 1958 and those who did not attend secondary high school. The lower mortality rate and the knowledge that both low educational level and immigration are associated with less favourable health [11,12], could indicate that the general health of the SPAF cohort is slightly better compared to the general Swedish population. However, compared to the general population, the participants in the SPAF cohort had similar aerobic capacity [13], physical activity [14], smoking habits [14], and level of overweight [14], compared to studies in general. The SPAF cohort can therefore be considered a fair representation of men and women born in Sweden in 1958.

## Data collection

At the ages 16, 27, 34 and 52, the testing took place in (or nearby) the original study cities. The procedures of these follow-ups have been described elsewhere [4,6,7,15].

At age 63, most participants came to Karolinska University Hospital for a full day of testing. The data collection period took place from March 2021 to July 2022. All baseline participants received a letter (and reminders if necessary) with an invitation for testing at the university clinic in Stockholm for a full day. The study was run during the covid pandemic, so those who did not want to travel were tested in one of the other five original cities.

To encourage both physically inactive and active, as well as unhealthy and healthy people to participate at age 63, we set up a web page to ensure that information about the study objectives and procedures were easily accessible to the cohort. We also made efforts to ensure that the participants´ visit to the clinic would be as pleasant and rewarding as possible. After discussions with the participants who came for testing, it was found that most of them did not feel the time commitment was too great, but rather felt they had an enjoyable and interesting day. Also, the prospect of having a health checkup, including neurography, cardiovascular examinations and blood health status might have influenced those who were willing to participate in the SPAF-1958 study. Efforts were made to get information about background, health, and lifestyle by questionnaire also from the cohort members who did not want to participate in testing. This information was gathered from a total of 59% of the original study cohort at age 63. However, the remaining eligible study cohort did not respond to repeated invitations or declined to participate. Unfortunately, only information on baseline characteristics is available for this part of the cohort.

## Methods

The tests for measuring physical capacity at baseline were selected to represent various qualities of physical fitness, as aerobic capacity, muscular endurance, and power. At age 63 the participants in the cohort have reached the age where age related loss of function starts to appear. This gives the opportunity to study the mechanisms behind the age-driven muscular wasting (sarcopenia). Therefore, the chair stand test and the handgrip tests were included as baseline for future follow-ups. These tests are used to investigate and assess probable sarcopenia [16]

Observed anthropometric and physical capacity test for all follow-ups are presented in Table S3 and S4. Observed muscle strength test related to identification of sarcopenia at age 63 are presented in Table S5.

## Study design

In this study, physical capacity was objectively measured repeatedly, and detailed information about the participants' lifestyle, performance and diagnoses was collected during 47 years of follow-up. One of the researchers was involved in preparing for the data collection at all follow-ups, ensuring that the tests were conducted in the same way at all follow-ups.

The study included tests of aerobic capacity, muscular endurance and power, as good physical capacity correlates with health outcomes [17,18]. The submaximal ergometer test and the Sargent jump tests are recognised and valid field tests [19–21] with acceptable reliability [22–24] and therefore suitable for studying the lifetime changes in physical capacity. The bench press test is unique to the SPAF study [25]; however, the test is similar in its administration to the YMCA bench press [26] which reliably assesses muscular endurance of the upper body.

One limitation of present study is that muscular power was not measured directly and was therefore estimated by jump height. However, jump height can be considered an approximative measure of jump power per kg body weight [27,28].

## Statistical analyses in the main paper

All statistical analyses were performed in R version 3.2 [29]. Linear mixed effect models were fitted **by maximum likelihood estimation (for the model fitting procedure comparing Akaike Information Criterion (AIC), and restricted maximum likelihood estimation (for the final models that are presented)** using the lmer function in the package lme4 [30]. Transformations were performed using the powerTransform function in the package car [31], natural splines were calculated using the ns function in the package splines [29] and differences in the predicted slope of age at different ages were tested using the emtrends function in the emmeans package [32].

Before analysis, residuals from random intercept models (eq 1) with fixed factors for sex and age and the age-by-sex interaction were analysed to find a transformation of the dependent variable (y) based on λ and γ, where λ=0 (log) was preferred over arbitrary values of λ if the profile log likelihood test of normality with λ=0 (log) indicated p=1:

### Linear mixed effect models

The base model equation is presented below (eq 1) and describe a random intercept (u) model with fixed effects for a function of age (*β*1), sex (*β2)* and the age-by-sex interaction (*β3),* where *y* is the dependent variable*,* β0 represent the (fixed effect) constant, e is the error term and subscripts indicate subjects (i) and observations (j) within subjects (eq 1). The model was also expanded to a random coefficient model by including a second random effect of age (eq 2).

*y_ij_* =*β*0 +*β*1**f*( age*_ij_* )+*β*2*sex*_ij_* +*β*3**(f*( age*_ij_* )*sex*_ij_* ) + *u_i_* +*e_ij_* _(eq 1)_

*y_ij_* =*β*0 +*β*1**f*( age*_ij_* )+*β*2*sex*_ij_* +*β*3**(f*( age*_ij_* )*sex*_ij_* )+*u0_i_* +*u1_i_* *** age*_ij_* +*e_ij_* _(eq 2)_

The intraclass correlation coefficient (ICC) was calculated from the random intercept model (eq 1) as the proportion of total error variance accounted for by the random intercept.

ICC=Var(u)/(Var(u)+Var(e)) (eq 3)

### Transformation of dependent variables

Before analysis, residuals from random intercept models (eq 1) with fixed factors for sex and age and the age-by-sex interaction were analysed to find a transformation of the dependent variable (y) based on λ and *γ*, where λ=0 (log) was preferred over arbitrary values of λ when the profile log likelihood test of normality for the log transformed variable indicated p=1. This resulted in a log transformation (λ=0) of all performance variables in this study:

z=0.5(y + (y^2+ *γ*^2)^0.5);

If λ=0, return log(z);

If λ>0, return z^λ;

If λ<0, return −z^λ. (eq 4)

### Model fitting procedure

*Factorial age models*: The main hypotheses of sex differences, effect of age and the age-by-sex interaction were tested using eq 1. In these models, sex and age was treated as categorical variables (factors) using orthogonal contrasts (sum to zero contrasts for sex and polynomial contrasts for age). Kenward-Roger's type-3 F-tests were used to test main effects and interactions for significance, with p<.05 considered statistically significant.

*Optimised models:* The model fit of the factorial models, calculated as the AIC, were compared to the fit of alternative models. These were models applying a linear function of age or a natural spline function of age with 2 degrees of freedom. All possible locations (in whole years) of the spline knot were tested to find the optimal shape of the spline function, and models were fit with and without an interaction sex*f(age), adjusting the intercept to the centre of age, to the beginning of the data collection at age 16 or a birth, with and without a random coefficient for age (eq 1 & 2). For each dependent variable, the model with the best fit (i.e. the lowest Akaike information criterion) was selected and used to plot model predictions and to test for differences in slope between the sexes (Figure S2)

*Lifestyle models:* The best fitted models were also used as baseline models to test hypotheses that theoretical achievement (university education) and physical activity (LTPA) could add to the prediction of the outcomes, above that of sex, age and the age-by-sex interaction. Two different models were fitted:

1. To estimate potential effects of early lifestyle choices on the outcomes, LTPA (at 16 years of age) and university education (at 34 years of age) were added with orthogonal contrast, as main effects and as interactions with age (Figure S3).

2. LTPA measured across the lifespan was added as the subject specific mean of all repeated measurements, and as a change from the subject specific mean, to separate between subject differences from within subject effects (change) on the outcomes (Figure S4).

### Individual differences

Predicted fixed effects together with subject specific trajectories based on both fixed and random effects were calculated. In all cases, the random coefficient model (eq 2), that allows individual differences to change across the lifespan, fitted data significantly better (p<.001) than the random intercept model (eq 1), assuming individual differences to be constant. Predicted random effects on the transformed variable were used for analysis

### Strengths and limitations with the statistical modelling

The longitudinal design of our study provides several advantages. Our results are derived using a statistical model which includes all the original participants. In longitudinal studies, systematic dropout may occur, but in the present study, statistical modelling made it possible to predict the decline in physical capacity for the entire baseline cohort. The use of linear mixed effects models allowed for unbiased estimation of model parameters under more relaxed assumptions of data missing at random (MAR), where the probability of missing data can depend on covariates in the model, rather than the more commonly assumed missing completely at random (MCAR) [33].

The reported effects of age describe changes within the same subjects rather than differences between groups of subjects from different generations that may exhibit other differences in e.g. behaviour, education, diet and healthcare. It allows for estimation of individual trajectories over time, based on random effects, and makes it possible to observe changes of individual differences in performance across the lifespan (e.g. variance increases). Also, the study design allows for the estimation of intraclass coefficient, providing a direct measure of the stability of individual differences in performance over the lifetime.

While the main hypotheses of sex, age and age-by-sex interaction differences were tested in factorial models, using discrete age categories defined by the data collection, more parsimonious models were developed that applied continuous functions of age based on natural splines. This made it possible to model inverted U-shape functions to predict performance between data collections and estimate approximate peak performance ages for the different tests as well as evaluate differences in slope between the sexes at all ages. However, while these predictions provide plausible estimates of performance as a function of age, they are mere approximations of the true functions, and the exact shape of performance across the life span needs to be verified in other studies, applying a higher data collection frequency at all ages, especially close to peak performance.

## Figure legends

**Figure S1**. Flow chart showing the part of the population participating at baseline and follow-ups.

**Figure S2.** Log transformed variables: Physical capacity from 16 to 63 years of age. Line charts represent estimated values for the entire baseline cohort with 95% CI (shadowed area). Dots represent observed values and SD. Lines on the x-axis indicate a sex difference in slope in relative performance.

**Figure S3**. Log transformed variables: Effect on physical capacity of being physically active at age 16 or having a university degree at any age. Line charts represent estimated values for the entire baseline cohort with 95% CI (shadowed area).

**Figure S4.** Log transformed variables: Effect on physical capacity of being physically active at age 16 or having a university degree at any age. Line charts represent estimated values for the entire baseline cohort with 95% CI (shadowed area).

# References

[1] Barnekow-Bergkvist M, Hedberg G, Janlert U, Jansson E. Prediction of physical fitness and physical activity level in adulthood by physical performance and physical activity in adolescence - An 18-year follow-up study. Scand J Med Sci Sports 1998;8:299–308. https://doi.org/10.1111/j.1600-0838.1998.tb00486.x.

[2] Hedberg G, Jansson E. Skelettmuskelfiberkomposition, kapacitet och intresse för olika fysiska aktiviteter bland elever i gymnasieskolan [Skeletal muscle fibre distribution, capacity and interest in different physical activities among pupils in high school]. 1976.

[3] Jansson E, Hedberg G. Skeletal muscle fibre types in teenagers: relationship to physical performance and activity. Scand J Med Sci Sports 1991;1:31–44. https://doi.org/10.1111/j.1600-0838.1991.tb00268.x.

[4] Glenmark B, Hedberg G, Jansson E. Changes in muscle fibre type from adolescence to adulthood in women and men. Acta Physiol Scand 1992;146:251–9. https://doi.org/10.1111/J.1748-1716.1992.TB09414.X.

[5] Barnekow-Bergkvist M, Hedberg G, Janlert U, Jansson E. Prediction of physical fitness and physical activity level in adulthood by physical performance and physical activity in adolescence - An 18-year follow-up study. Scand J Med Sci Sports 1998;8:299–308. https://doi.org/10.1111/j.1600-0838.1998.tb00486.x.

[6] Aasa U, Lundell S, Barnekow-Bergkvist M, Jansson E, Westerståhl M. The Swedish physical activity and fitness cohort born in 1958 - dropout analysis and overview at 36-year follow-up. Scand J Med Sci Sports 2017;27:418–29. https://doi.org/10.1111/sms.12665.

[7] Barnekow-Bergkvist M, Hedberg G, Janlert U, Jansson E. Development of muscular endurance and strength from adolescence to adulthood and level of physical capacity in men and women at the age of 34 years. Scand J Med Sci Sports 1996;6:145–55.

[8] Statistics Sweden. Statistiska meddelanden-Befolkningens utbildning 2020. 2020.

[9] Statistics Sweden. Summary of Population Statistics 1960–2019 n.d.

[10] Statistics Sweden. Döda, antal efter region, ålder, kön och år. PxWeb n.d.

[11] Östergren O. Educational inequalities in mortality are larger at low levels of income: A register-based study on premature mortality among 2.3 million Swedes, 2006–2009. SSM Popul Health 2018;5:122–8. https://doi.org/10.1016/j.ssmph.2018.05.008.

[12] Helgesson M, Johansson B, Nordquist T, Vingård E, Svartengren M. Healthy migrant effect in the Swedish context: A register-based, longitudinal cohort study. BMJ Open 2019;9. https://doi.org/10.1136/bmjopen-2018-026972.

[13] Väisänen D, Ekblom B, Wallin P, Andersson G, Ekblom-Bak E. Reference values for estimated VO2max by two submaximal cycle tests: the Åstrand-test and the Ekblom-Bak test. Eur J Appl Physiol 2024;124:1747–56. https://doi.org/10.1007/s00421-023-05398-8.

[14] Folkhälsomyndigheten. Öppna jämförelser – Folkhälsa 2019 [Internet]. Solna: Folkhälsomyndigheten; [cited 2025 Apr 13]. Available from: https://www.folkhalsomyndigheten.se/contentassets/ec714fca0b0145eab3d7924511550a74/oppna-jamforelser-folkhalsa-2019-18076.pdf. 2019.

[15] Barnekow-Bergkvist M, Hedberg G, Janlert U, Jansson E. Physical activity pattern in men and women at the ages of 16 and 34 and development of physical activity from adolescence to adulthood. Scand J Med Sci Sports 1996;6:359–70.

[16] Cruz-Jentoft AJ, Bahat G, Bauer J, Boirie Y, Bruyère O, Cederholm T, et al. Sarcopenia: revised European consensus on definition and diagnosis EUROPEAN WORKING GROUP ON SARCOPENIA IN OLDER PEOPLE 2 (EWGSOP2), AND THE EXTENDED GROUP FOR EWGSOP2. Age Ageing 2019;48:16–31. https://doi.org/10.1093/ageing/afy169.

[17] Singh H, Kim D, Kim E, Bemben MG, Anderson M, Seo D Il, et al. Jump test performance and sarcopenia status in men and women, 55 to 75 years of age. Journal of Geriatric Physical Therapy 2014;37:76–82. https://doi.org/10.1519/JPT.0b013e3182a51b11.

[18] Korpelainen R, Lämsä J, Kaikkonen KM, Korpelainen J, Laukkanen J, Palatsi I, et al. Exercise capacity and mortality – a follow-up study of 3033 subjects referred to clinical exercise testing. Ann Med 2016;48:359–66. https://doi.org/10.1080/07853890.2016.1178856.

[19] Markovic G, Dizdar D, Jukic I, Cardinale M. Reliability and factorial validity of squat and countermovement jump tests. J Strength Cond Res 2004;18:551–5. https://doi.org/10.1519/1533-4287(2004)18<551:RAFVOS>2.0.CO;2.

[20] Väisänen D, Ekblom Ö, Ekblom-Bak E, Andersson E, Nilsson J, Ekblom M. Criterion validity of the Ekblom-Bak and the Åstrand submaximal test in an elderly population. Eur J Appl Physiol 2020;120:307–16. https://doi.org/10.1007/s00421-019-04275-7.

[21] Teräslinna P, Ismail AH, MacLeod DF. Nomogram by Astrand and Ryhming as a predictor of maximum oxygen intake. J Appl Physiol 1966;21:513–5. https://doi.org/10.1152/jappl.1966.21.2.513.

[22] Markovic G, Dizdar D, Jukic I, Cardinale M. Reliability and factorial validity of squat and countermovement jump tests. J Strength Cond Res 2004;18:551–5. https://doi.org/10.1519/1533-4287(2004)18<551:RAFVOS>2.0.CO;2.

[23] Hedberg G. Pedagogiska rapporter nr 50. Utprövning av styrketest för skolelever (A try-out of tests of strength for pupils). Umeå: 1975.

[24] Phillips BE. The jcr test. Research Quarterly of the American Association for Health, Physical Education and Recreation 1947;18:12–29. https://doi.org/10.1080/10671188.1947.10620951.

[25] Hedberg G. A try-out of tests measuring strenght in school children [Utprövning av styrketest för skolelever] in Swedish. 1975.

[26] Golding LA, Myers CR, Sinning WE. The Y’s way to physical fitness : A guide book for instructors (Rev. ed.), YMCA of the USA 1982.

[27] Markovic G, Jaric S. Is vertical jump height a body size-independent measure of muscle power? J Sports Sci 2007;25:1355–63. https://doi.org/10.1080/02640410601021713.

[28] Siglinsky E, Krueger D, Ward RE, Caserotti P, Strotmeyer ES, Harris TB, et al. Effect of age and sex on jumping mechanography and other measures of muscle mass and function. Journal of Musculoskeletal Neuronal Interactions 2015;15:301–8.

[29] R Core Team. R: A Language and Environment for Statistical Computing 2023. https://doi.org/https://www.R-project.org/.

[30] Bates D, Mächler M, Bolker BM, Walker SC. Fitting linear mixed-effects models using lme4. J Stat Softw 2015;67. https://doi.org/10.18637/jss.v067.i01.

[31] Fox J, Weisberg S. An R Companion to Applied Regression. 3rd ed. Thousand Oaks CA: Sage; 2019.

[32] Lenth R. emmeans: Estimated Marginal Means, aka Least-Squares Means_. R package version 1.10.0 2024.

[33] Schafer JL, Graham JW. Missing data: Our view of the state of the art. Psychol Methods 2002;7:147–77. https://doi.org/10.1037/1082-989X.7.2.147.
