# Supplementary material for: Rise and Fall of Physical Capacity in a General Population: A 47‐Year Longitudinal Study
Source: J Cachexia Sarcopenia Muscle. 2025 Nov 16;16(6):e70134. doi: 10.1002/jcsm.70134 (PMC12620399; doi:10.1002/jcsm.70134)
Supplement: Supplementary file 8 — Data S2: Supplementary Information. [file JCSM-16-e70134-s007.docx]

1. Barnekow-Bergkvist M. Hedberg G. Janlert U. Jansson E. Development of muscular endurance and strength from adolescence to adulthood and level of physical capacity in men and women at the age of 34 years. Scand J Med Sci Sports 1996;6:145–55. <https://doi.org/10.1111/j.1600-0838.1996.tb00082.x>.
2. Barnekow-Bergkvist M. Hedberg G. Janlert U. Jansson E. Prediction of physical fitness and physical activity level in adulthood by physical performance and physical activity in adolescence--an 18-year follow-up study. Scand J Med Sci Sports 1998;8:299–308.
3. Barnekow-Bergkvist M. Hedberg GE. Janlert U. Jansson E. Health status and health behaviour in men and women at the age of 34 years. Eur J Public Health 1998;8:179–82. <https://doi.org/10.1093/eurpub/8.2.179>.
4. Barnekow-Bergkvist M. Hedberg G. Pettersson U. Lorentzon R. Relationships between physical activity and physical capacity in adolescent females and bone mass in adulthood. Scand J Med Sci Sports 2006;16:447–55. <https://doi.org/10.1111/j.1600-0838.2005.00500.x>.
5. Glenmark B. Hedberg G. Jansson E. Changes in muscle fibre type from adolescence to adulthood in women and men. Acta Physiol Scand 1992;146:251–9. <https://doi.org/10.1111/j.1748-1716.1992.tb09414.x>.
6. Barnekow-Bergkvist M. Hedberg G. Janlert U. Jansson E. Physical activity pattern in men and women at the ages of 16 and 34 and development of physical activity from adolescence to adulthood. Scand J Med Sci Sports 1996;6:359–70. <https://doi.org/10.1111/j.1600-0838.1996.tb00108.x>.
7. Bmi ISO. Flickor 2014;7:2012–4.
8. Hedberg G. Pedagogiska rapporter. Utprövning av ett uthållighetsprov för skolelever (A try-out of a test of endurance for pupils). Umeå: 1975.
9. Hedberg G. Jansson E. Skelettmuskelfiberkomposition. kapacitet och intresse för olika fysiska aktiviteter bland elever i gymnasieskolan [Skeletal muscle fibre distribution. capacity and interest in different physical activities among pupils in high school]. 1976.
10. Åstrand I. Aerobic work capacity in men and women with special reference to age. Acta Physiol Scand Suppl 1960;49:1–92.
11. Bergkvist M. Hedberg G. Rahm M. [Utvärdering av test för bedömning av styrka. rörlighet och koordination] in Swedish. Solna. Sweden: Arbetsmiljöverket; 1992.
12. Sargent DA. The Physical Test of a Man. American Physical Education Review 1921;26:188–94. <https://doi.org/10.1080/23267224.1921.10650486>.
13. Markovic G. Jaric S. Is vertical jump height a body size-independent measure of muscle power? J Sports Sci 2007;25:1355–63. <https://doi.org/10.1080/02640410601021713>.
14. Siglinsky E. Krueger D. Ward RE. Caserotti P. Strotmeyer ES. Harris TB. et al. Effect of age and sex on jumping mechanography and other measures of muscle mass and function. Journal of Musculoskeletal Neuronal Interactions 2015;15:301–8.
15. R Core Team. R: A Language and Environment for Statistical Computing 2023. <https://doi.org/https://www.R-project.org/>.
16. von Haehling S. Coats AJS. Anker SD. Ethical guidelines for publishing in the Journal of Cachexia. Sarcopenia and Muscle: update 2021. J Cachexia Sarcopenia Muscle 2021;12:2259–61. https://doi.org/10.1002/jcsm.12899.
